# Alcohol and the Human Body

**Published:** 1919-12-06

**Authors:** 


### ALCOHOL AND THE HUMAN BODY.

### Some Recent Experiments Upon its Action.

At the request of the Centrol Control Board (Liquor Traffic) this most interesting work was undertaken by Dr. Edward Mellanby, whose services were lent, together with the provision of the necessary expenses, by the Medical Research Committee, and the report recently published by H.M. Stationery Office, arising from an investigation commencing under such auspices, is particularly remarkable for its direct reference to many practical issues. The primary aim was to decide how far the circumstances in which a given amount of alcohol was consumed could affect the degree of intoxication which it produced.

At the outset criticism may arise owing to the fact that Dr. Mellanby has been confined to the use of dogs as subjects, and there arises the obvious difficulty of estimating or expressing the intensity of intoxication as shown by an animal in any terms which can be applied with certainty to a human being. A very satisfactory variable was, however, considered and estimated—namely, the concentration of alcohol in the blood found at known periods after giving a variety of doses by the mouth. The author justifiably assumes that the factors shown to affect the results in a dog are likely to have a corresponding influence in man, and, as stated in the introduction, it is now learnt that preliminary experiments already carried out on human beings suggest that the results are strictly comparable.

### The Main Conclusions.

In the first place, it is found that the maximal concentration in the blood, which occurs very soon after the alcohol is consumed, is, within certain limits, proportional to the amount actually taken. Proceeding further, we come to another vital fact. In all the observations described it may be noted that the subsequent disappearance of the alcohol from the blood is a remarkably slow process, with the result that, whether a given amount of spirit be taken in one dose or in divided doses at intervals extending even to two hours, the *same* maximal concentration is produced. In other words, the effect is here cumulative, but, if the actual spirit be diluted before administration, then the alcohol usually accumulates in the blood more slowly, producing a lower maximum than before and giving experimental proof and explanation of the observed fact that, the weaker the beverage, the more the actual alcohol which must be consumed in order that a definite degree of intoxication will be reached.

On the other hand, another effect of dilution may occur. If the water or other fluid be taken a few hours before a dose of alcohol, the absorption of the latter is much more rapid and leads consequently—for a given dose—to a far greater blood concentration. Foodstuffs, however, are found to delay intoxication by delaying absorption from the alimentary canal. The most effective foodstuff in this respect is milk. Its specific influence in delaying absorption more than counter-balances its general effect as a fluid, and Dr. Mellanby comments upon the striking differences observed in the effects of a dose of alcohol when given two hours after the consumption of half a litre of water, and half a litre of milk respectively. In the first case a dog may become incapable of standing or walking, in the latter case it may show no signs whatever of unsteadiness.

One scientific point stands out as particularly well established by these experiments and is of great interest. Alcohol disappears from the circulation with remarkable slowness, and at a rate which is independent of its concentration in the blood stream.

### Intoxication and Thirst.

In spite of the conclusions already given one notes that it is exceedingly difficult to relate the actual absorption of alcohol to the sense of thirst, in that the latter is so markedly influenced by the varying conditions of the alimentary canal, but, although it is impossible here to describe the more detailed evidence, it seems very likely that, since alcohol needs no digestion for absorption into the blood stream, some other factor besides the stimulating effect of draughts of water on the gastric secretion, as proved by Pawlow and others, must be called into play. Possibly there is a specific stimulation of the intestinal mucous membrane responsible for absorption, and the fact that the water effect continues even after four hours suggests that its action may be on these lines, or even a washing away from the walls of the alimentary canal of some substance normally tending to inhibit absorption. The occurrence of this acceleration of alcoholic effects is, however, definitely established, be the actual reason what it may, and it is interesting to realise therefrom that a first drink of a dilute alcoholic beverage may thus act as a marked stimulant to the absorption of the alcohol from later ones. The practical application is noted in the following conclusion. In order to obtain alleviation of symptoms of intoxication by splitting up an alcoholic beverage into two or three portions the intervals between the drinks must be long, three or more hours, the time depending on the concentration of the fluid and the amount of alcohol contained therein. It is probable that the more dilute the beverage and the fewer the portions into which the drink is divided, the larger must be the interval between drinking in order to escape the maximum intoxicating symptoms.

### Alcohol as a Food.

On this vexed question Dr. Mellanby's experiments throw considerable light, and the evidence is on the whole most unfavourable to those who

**Alcohol and the Human Body**—(*continued*).

would unduly support the value of alcohol in this respect. In the first place its rate of combustion is apparently independent of the amount of circulating blood within fairly wide limits. Also it has no specific dynamic action, but simply replaces other foodstuffs without stimulating the total combustion processes, and the rate of combustion is decidedly independent of the types of other foodstuffs being metabolised.

The result of exercise on this combustion rate is also dealt with, and here clear indication arises that whereas alcohol, when present in the body in low concentration undergoes a more rapid breakdown during exercise and therefore supplies a greater amount of energy, at higher concentrations the combustion rates are the same in both the resting and active animals. "In other words, the greater the toxic action of the alcohol, the more limited is the increase in its rate of combustion by exercise, and the closer do the rates of combustion of alcohol in the active and resting states approximate. Alcohol at high concentration seems not only to have this self-limiting action on its own oxidative process, but, if fatigue is the true indication of diminished or partial oxidation, to extend its baneful and detrimental influence to the limitation of the oxidation of other combustible material."

Many of these results and conclusions confirm ideas which have of late found fairly general acceptance, in others there is definite indication of points which have been very commonly missed, and thus, when considering the habitual use of spirits, the report may be read with greatest benefit by all concerned with these matters from either a clinical or an administrative point of view.

